# A Single-Cell Transcriptome of Bovine Milk Somatic Cells

**DOI:** 10.3390/genes15030349

**Published:** 2024-03-10

**Authors:** Minja Zorc, Mateja Dolinar, Peter Dovč

**Affiliations:** Department of Animal Science, Biotechnical Faculty, University of Ljubljana, Jamnikarjeva 101, 1000 Ljubljana, Slovenia; minja.zorc@bf.uni-lj.si (M.Z.); mateja.dolinar@bf.uni-lj.si (M.D.)

**Keywords:** mammary gland, scRNA-seq, lactation, cattle, transcriptome, milk somatic cells, differential gene expression

## Abstract

The production of milk by dairy cows far exceeds the nutritional needs of the calf and is vital for the economical use of dairy cattle. High milk yield is a unique production trait that can be effectively enhanced through traditional selection methods. The process of lactation in cows serves as an excellent model for studying the biological aspects of lactation with the aim of exploring the mechanistic base of this complex trait at the cellular level. In this study, we analyzed the milk transcriptome at the single-cell level by conducting scRNA-seq analysis on milk samples from two Holstein Friesian cows at mid-lactation (75 and 93 days) using the 10× Chromium platform. Cells were pelleted and fat was removed from milk by centrifugation. The cell suspension from each cow was loaded on separate channels, resulting in the recovery of 9313 and 14,544 cells. Library samples were loaded onto two lanes of the NovaSeq 6000 (Illumina) instrument. After filtering at the cell and gene levels, a total of 7988 and 13,973 cells remained, respectively. We were able to reconstruct different cell types (milk-producing cells, progenitor cells, macrophages, monocytes, dendritic cells, T cells, B cells, mast cells, and neutrophils) in bovine milk. Our findings provide a valuable resource for identifying regulatory elements associated with various functions of the mammary gland such as lactation, tissue renewal, native immunity, protein and fat synthesis, and hormonal response.

## 1. Introduction

The mammary gland is a relatively recent acquisition of mammalian evolution, essential for successful reproduction, as it provides nourishment and immune protection for the neonate in the first weeks of life. The mammary gland is a highly regenerative organ and one of the few tissues that undergo most of its development after birth [1]. The cyclical phases of growth, differentiation, lactation, and involution of the mammary gland are regulated by hormones and growth factors [2].

A consequence of this complex function of the mammary gland and intense secretion of milk, which differs significantly among different species, is also the presence of somatic cells in milk. The main fractions of somatic cells in milk are epithelial cells, lymphocytes, polymorphonuclear neutrophils (PMN), and macrophages. The majority of exfoliated epithelial cells present in milk are viable and exhibit characteristics of fully differentiated alveolar cells [3]. Since the somatic cell count (SCC), widely used as a marker for udder health, only provides the cumulative number of somatic cells in milk, allows the newly developed differential somatic cell count (DSCC) differentiation between two groups of cells: PMN and lymphocytes versus macrophages [4]. Therefore, differential somatic cell count represents a significant step forward in understanding the dynamics of the somatic cell population in the mammary gland during lactation and at infection. In cattle and sheep, the epithelial cell fraction represents only a relatively small part of somatic cells in milk, whereas, in porcine milk, similar to human milk, epithelial cells are the predominant cell type in milk [3].

In diverse organs, adult stem cells are present with their primary role of maintaining tissue homeostasis [5]. However, stem cells in the adult mammary gland serve both development and homeostasis. Mammary stem cells (MaSCs) can self-renew and differentiate into different cell types during the mammary gland’s developing cycles [6]. Because of dramatic changes observed in mammary epithelium during morphogenesis and the reproductive cycle, researchers have for many years suspected the existence of mammary stem cells. In the 1950s, fat pad transplantation studies in mice demonstrated the regenerative and differentiation capacity of epithelial mammary gland cells [7,8]. In 2006, it was reported that mouse MaSCs were identified and isolated [9]. Since then, plenty of strategies, such as transplantation of tissue, colony-forming assays, cell populations sorted for cell surface markers, and lineage tracing, have been used to identify and characterize MaSC and to delineate the mammary epithelial hierarchy [1].

In order to gain an insight into the molecular events in the lactating mammary gland at the cellular level, access to the relevant biological material is required. Taking biopsies from the mammary gland is one possibility to obtain material for different types of studies. However, due to its invasive nature, researchers were looking for alternatives to biopsies. The comparison of five different sources of RNA (biopsies of the mammary gland tissue, laser microdissected mammary epithelial cells, milk somatic cells, milk fat globules, and antibody-captured milk mammary epithelial cells) for analysis of the bovine mammary gland transcriptome, showed that isolation of total RNA directly from somatic milk cells released into milk during lactation is an effective alternative to mammary gland tissue biopsies and laser microdissection of mammary epithelial cells [10].

The first data about organ-specific gene expression in the mammary gland were obtained using expression microarrays a decade ago [11]. This approach revealed the expression of genes involved in cell development, growth, proliferation, and cell morphology in the human milk cells from milk fat globules. In addition, it allowed a comparative approach between species but was limited by the selection of genes on the chip. The next important step represented bulk RNA sequencing from mammary gland isolates [12]. Sequencing of bulk RNA isolated from bovine milk cells in three lactation stages, transition lactation (day 15), peak lactation (day 90), and late lactation (day 250) in Holstein cows revealed expressions of 16,892, 19,094, and 18,070 genes, respectively. This is a cumulative number of genes expressed in different cell types present in cow’s milk. Independent of the lactation stage, approximately 9000 genes showed ubiquitous expression, genes encoding caseins, whey proteins, and enzymes in the lactose synthesis pathway showed higher expression in early lactation, and also the majority of genes in the fat metabolism pathway had high expression in transition and peak lactation [13].

It has recently become possible to analyze the transcriptomes of single cells [14]. Since then, single-cell RNA sequencing of bovine milk cells has been performed and revealed immune (macrophages, monocytes, dendritic cells, T cells, B cells, and NK cells) and epithelial cells, 2.47% of the cells were epithelial cells [15]. Single-cell transcriptomic studies on human milk have shown it predominantly comprises epithelial cells from the luminal lineage. Endothelial and immune cells make up approximately 1.4 ± 1.1% of the total cell count in mature human milk samples. Furthermore, two distinct secretory cell types were identified in human milk [16]. The study of mammary epithelial cells at the single-cell level in humans and mice revealed much greater heterogeneity in the composition of mammary epithelial cells than previously reported [17]. A few studies have examined mammary gland cells from mice and humans, analyzing the epithelial cells of the mammary gland [18,19,20,21,22,23] or the entire mammary gland at the single-cell level [24,25]. The analyses revealed several different cell types. The researchers reported mostly consistent, but also some discordant results, possibly due to differences in the methods of analysis.

The capacity of milk production in dairy cows exceeds several times the nutritional needs of the calf and is essential for the economical use of dairy cattle in agriculture. Lactation in cattle is a suitable model for studying the biology of lactation with the aim of discovering the mechanistic base of this complex trait at the cellular level. The single-cell RNA sequencing opens a new horizon for documentation of cell type-specific expression profiles in the mammary gland and even the possibility of determining different cell types based on cell type-specific transcriptomic profiles [20]. This approach also allows the identification of cellular sources for several milk components, which did not have a defined origin before [26].

Milk contains mammary epithelial cells and immune system cells (lymphocytes, macrophages, and neutrophils), which reflect the activity of the mammary gland and reveal the response of the mammary gland to environmental challenges [27]. The gene expression of ductal, alveolar, and stromal cells in the mammary gland drives lactation and reveals the cyclic character of the mammary gland [28]. Here, we report the application of scRNA-seq to elucidate the cell type repertoire in bovine milk based on the transcriptomic differences between different cell clusters. We were focused on defining the resident cell types in mid-lactation bovine milk. This information will contribute to a better understanding of the complex processes in the mammary gland, including tissue remodeling and involution.

## 2. Materials and Methods

### 2.1. Bovine Milk Samples and Isolation of Cells

Milk samples were collected from two healthy Holstein Friesian cows in mid-lactation (75 and 93 days) from a dairy farm in Slovenia. The sample collection was performed in February and animals were on a standard grass/maize silage diet with the addition of a standard concentrate for dairy cows. A regular milking test was performed five days prior to sampling and the records are as follows: 49.6 and 51.2 milk yield (kg), 3.46 and 3.08 fat (%), 3.37 and 3.22 protein (%), 4.71 and 4.77 lactose (%), 19,000 and 25,000 SSC, and 30 and 34 mg urea per liter. Milk samples were taken after morning milking with manual milking from all four quarters, 30 mL per quarter and 120 mL from each animal. Fresh milk samples were transported to the laboratory on ice and processed within three hours of being collected. The cells were pelleted and the fat was removed from the milk in 50-mL tubes by centrifugation (500× *g*) for 5 min. The supernatants were decanted and the pellets were pooled by resuspending them in approximately 5 mL of cold phosphate-buffered saline (PBS). The pellet was washed by removing the supernatant and resuspending the cells in 5 to 10 mL of cold PBS before transferring the sample to a new 15 mL tube and centrifuging at 490 *g* for 5 min at 4 °C. The ratio of dead cells was estimated by trypan blue (0.2%) staining (1:1) of cell suspension in the cell counting chamber.

### 2.2. Single-Cell Library Preparation and Sequencing

Single-cell library generation for 10X Genomics chemistry was performed following Chromium Single Cell 3′ Reagent Kits user guide (v3 Chemistry) [29]. In brief, cell suspensions were loaded onto a 10X Chromium Controller instrument (10x Genomics, Pleasanton, CA, USA) to generate single-cell GEMs for two biological replicates followed by cell lysis and barcoded reverse transcripts of RNA, amplification, shearing, and 5′ adapter and sample index attachment. Quality control and quantification of the resulting polymerase chain reaction (PCR) products were determined using a DNA high-sensitivity assay on a PerkinElmer-HP (Waltham, MA, USA). The peak of the fragment size distribution was around 1200 to 1500 bp indicating a good quality of cDNA synthesis (Figure S1).

Library samples were diluted to a concentration of 10 nM and loaded onto two lanes of the NovaSeq 6000 (Illumina, San Diego, CA, USA) instrument. Two samples (27.5 and 20.0 μL of cell suspension) were loaded on each channel which resulted in the recovery of 9313 and 14,544 cells. A total of ~361 million reads were obtained with 36,315 mean reads per cell for the first replicate and ~257 million reads with 17,459 mean reads per cell for the second replicate. For the first replicate, 96.6% of the reads were mapped to the genome and for the second replicate 94.1%. We detected a total of 15,630 and 16,497 genes, corresponding to 735 and 661 median genes per cell in the first and second biological replications, respectively.

### 2.3. Pre-Processing and Quality Control of scRNA-Seq Data

Sample demultiplexing, barcode processing, read alignment to the bovine reference genome (ARS-UCD1.2.108), quantification, and initial quality control of paired-end sequencing data were performed for each sample using Cell Ranger software (version 7.1.0, 10X Genomics). The sequencing saturation determined by Cell Ranger showed a saturation of 77.2% and 66.3%. The output of Cell Ranger was used for further processing with R (version 4.3.2) and the R package Seurat v4.2.0 [30]. Genes expressed in less than three cells were removed from the gene expression matrix. We kept the cells with a minimum of 200 and a maximum value of 2500 expressed genes. Cells in which mitochondrial genes accounted for more than 20% of the counts were filtered out. After filtering, 7988 and 13,973 cells remained.

### 2.4. Identification of Cell Clusters

We applied Seurat’s “anchor-based” workflow [31] to integrate two datasets. After filtering, we log-normalized the raw counts with *LogNormalize* and used *FindVariableFeatures* function to identify highly variable genes for each batch at default settings. We then ran *FindIntegrationAnchors* with dims = 1:30. The resulting anchors were used for *IntegrateData* with the 30 dimensions. We then scaled all genes with *ScaleData* and performed a principal component analysis with *RunPCA*, all with the default settings. Clusters were identified using the *FindClusters* function with a resolution of 0.8 and then visualized using the *RunTSN* and *RunUMAP* (reduction = “pca”) functions.

### 2.5. Annotation of Cell Types

For the fully automated identification of cell types, we used ScType [32] with the ScType marker database (https://www.nature.com/articles/s41467-022-28803-w, accessed on 18 September 2023) and clustermole: Unbiased Single-Cell Transcriptomic Data Cell Type Identification R package version 1.1.1. (https://igordot.github.io/clustermole/, accessed on 25 February 2024). We used singleCellBase, a manually curated database of cell markers for scRNA-Seq annotation [33]. Cell types were also manually assigned to cell clusters by matching cluster-specific upregulated marker genes with prior knowledge of cell type markers.

### 2.6. Identification of Highly Variable Expressed Genes

The most variable genes based on their expression in the whole population were determined using the *FindVariableGenes* function [31,34] with the default parameters (selection.method = “vst”, nfeatures = 2000). This function is used to calculate the average expression and dispersion for each gene. The genes are placed into bins and then the z-score for dispersion is calculated for each bin. We selected the 2000 genes with the highest standardized variance.

## 3. Results

### 3.1. ScRNA-Seq Identifies a Diversity of Cell Types in Bovine Milk Somatic Cells

**Figure 1:**
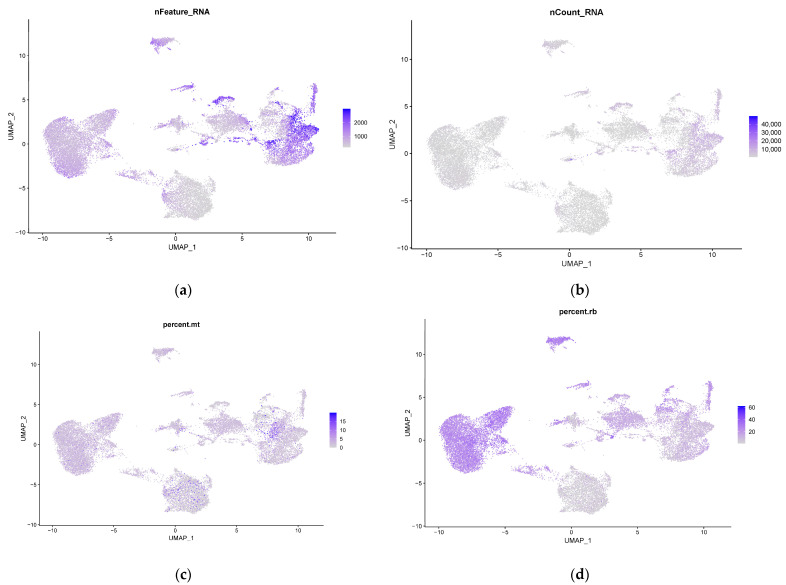
ScRNA−seq quality control analysis with UMAP plots of two somatic milk cell datasets after integration. The colors indicate: (**a**) number of genes per cell (nFeature_RNA), (**b**) number of UMI reads per cell (nCount_RNA), (**c**) percentage of cell counts mapping to mtDNA genes (percent.mt), and (**d**) percentage of cell counts mapping to ribosomal protein transcripts (percent.rb).

Transcriptional profiling of bovine somatic milk cells was performed using scRNA-seq analysis with the 10x Chromium platform on fresh milk samples from two Holstein Friesian cows in mid-lactation (75 and 93 days). Cell quality control was based on the number of genes per cell, the number of UMI reads per cell, the percentage of cell counts mapping to mtDNA genes, and the percentage of cell counts mapping to ribosomal protein transcripts. Bovine milk somatic cells have slightly different numbers of expressed genes per cell and regulation of mitochondrial and ribosomal transcripts (Figure 1). After filtering at cell and gene levels, a total of 7988 and 13,973 cells remained. Since we had two individual scRNA-seq samples, we conducted an anchor-based integration analysis to explore all cells in these samples simultaneously.

**Figure 2:**
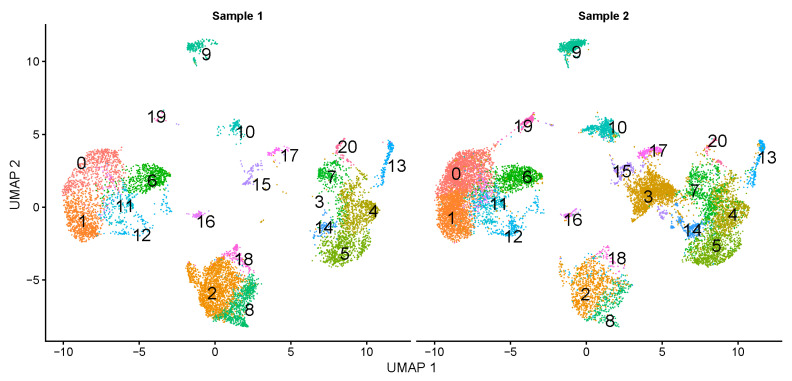
Side-by-side comparison of UMAP projections of cell clusters identified in two samples of fresh bovine milk developed from scRNA-seq. Cells were grouped into 21 matching clusters (labeled with 0 to 20) in Sample 1 and Sample 2.

After normalization and integration of the data, the cells from each of the two milk samples appeared to be evenly distributed along the cell projection (Figure 2). UMAP reduction generates a clear cell clustering highlighting 21 distinct cell populations based on their gene expression profiles. All identified clusters were shared by both samples (Figure 2).

**Figure 3:**
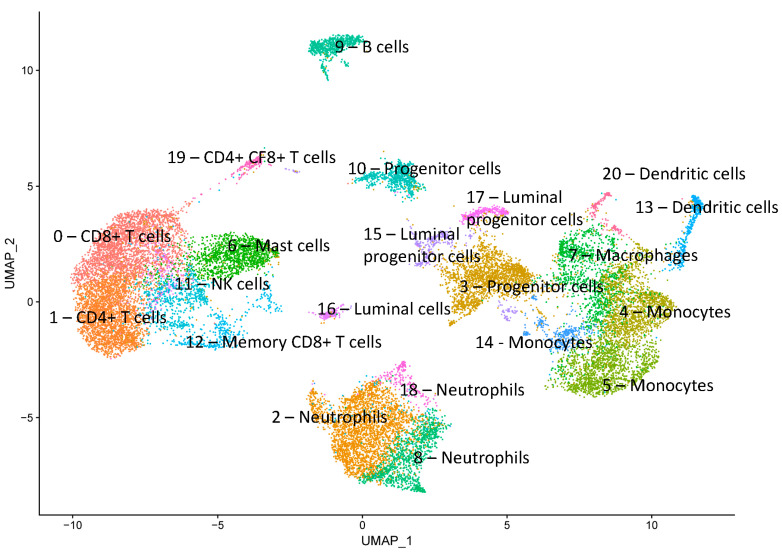
Annotated cell clusters (*n* = 21) from two bovine milk samples.

**Figure 4:**
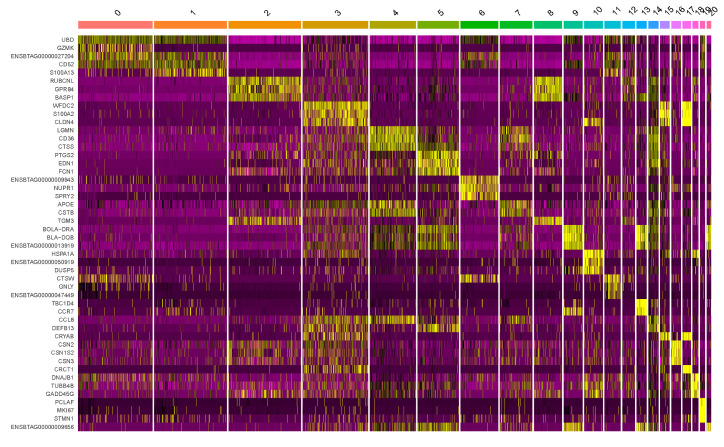
The specificity of marker gene expression was presented with a heatmap of standardized expression for the three major marker genes identified for each of 21 clusters of the cells. The genes are shown in rows and the individual cells in columns. High expression of a particular gene is labeled with yellow and low expression with purple.

**Figure 5:**
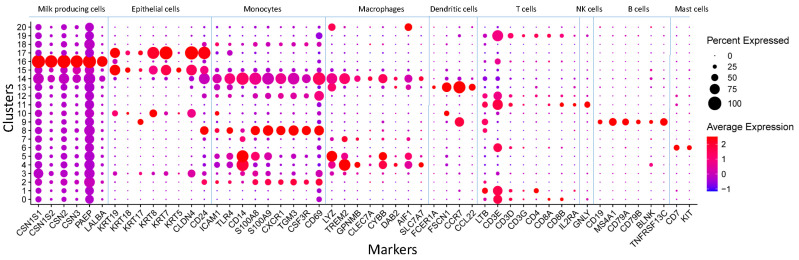
Dot plot highlighting the marker genes used to determine the cluster identities of 21 clusters. The marker genes are listed on the X axis and the cluster numbers are on the Y axis. The size of the circle corresponds to the number of cells in the cluster expressing the marker, while the shading corresponds to the extent of expression.

We identified 21 cell clusters and annotated them (Figure 3) as T cells (CD8+, CD4+), neutrophils, progenitor cells, monocytes, mast cells, macrophages, B cells, NK cells, dendritic cells, monocytes, luminal cells, and luminal progenitor cells. These annotations were determined using automated methods for cell type annotation and examination of established marker genes for each cell type as well as major marker genes for each cluster (Table 1, Figure 4 and Figure 5).

The cells responsible for milk production were identified in three clusters, 15, 16, and 17. These clusters were classified as luminal progenitor cells and luminal cells characterized by the expression of genes encoding caseins and whey proteins and epithelial markers. Clusters 3 and 10, which express epithelial markers, were identified as progenitor cells using the Clustermole tool, which found similar cell types in a database of mouse mammary gland cells (cluster 3 being hormone-sensitive progenitor cells and cluster 10 being other progenitor cells). Monocytes were identified in clusters 4, 5, and 14, characterized by the expression of monocyte markers (CD14). Cluster 7 contained macrophages, clusters 13 and 20 dendritic cells, and clusters 2, 18, and 8 neutrophils. T cells expressing either CD4, CD8, or both markers were located in clusters 0, 1, 12, and 19; NK cells were located in cluster 11, while B cells expressing specific marker genes (*CD19*, *MS4A1*, *CD79A*, *CD79B*, *BLNK,* and *TNFRSF13C*) formed cluster 9. Mast cells expressing genes CD7 and KIT were identified in cluster 6.

### 3.2. Milk Producing Cell Clusters

**Figure 6:**
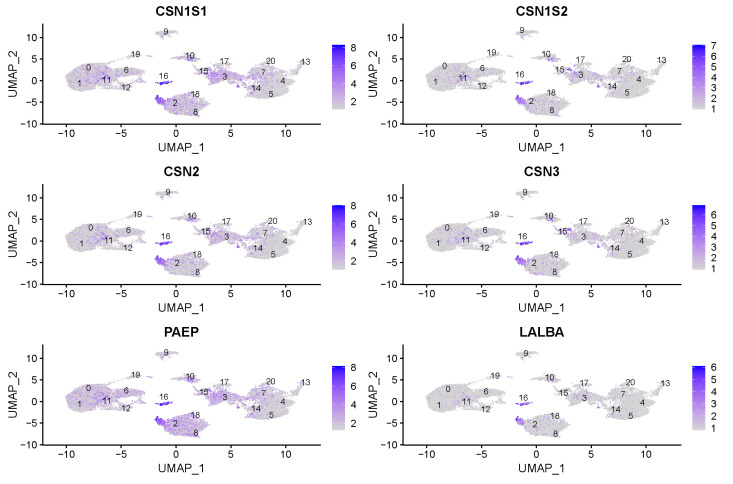
Expression of casein (*CSN1S1*, *CSN1S2*, *CSN2*, *and CSN3*) and whey protein (*PAEP* and *LALBA*) genes in bovine somatic milk cell clusters (labeled with 0 to 20).

To identify milk-producing cells, we examined the expression of casein (*CSN1S1*, *CSN1S2*, *CSN2*, *CSN3*) and whey protein (*PAEP*, *LALBA*) genes (Figure 6). In both samples, expression of milk-producing genes was present, *CSN1S1* was expressed in 50.2% of cells in the first and in 52.4% of cells in the second sample, *CSN1S2* was expressed in 22.4% and 21.2% of cells, *CSN2* was expressed in 53.0% and 38.2%, *CSN3* in 33.1% and 20.9%, *PAEP* in 82.0% and 66.3% and, *LALBA* in 21.3% and 12.6% of recovered cells. Significantly higher levels of caseins and whey proteins are detected in cluster 20, annotated as alveolar cells. However, caseins and whey proteins were expressed also in clusters 18 and 19 assigned as neutrophils and effector CD4+ T cells.

The expression of casein (*CSN1S1*, *CSN1S2*, *CSN2*, *and CSN3*) genes, whey protein (*PAEP* and *LALBA*) genes, *MUC15* and *BTNA1* genes shows cell-type specific profiles which indicate differences among bovine somatic milk cell clusters.

### 3.3. Highly Variable Expressed Genes in Bovine Milk Somatic Cells

**Figure 7:**
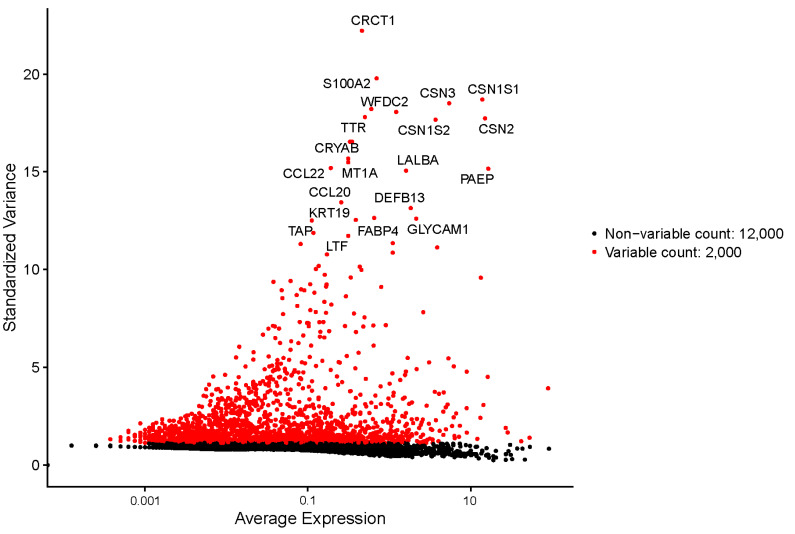
Twenty most highly variable expressed genes in bovine milk somatic cells are presented on standardized variance vs. average expression plot. Red dots indicate the differentially expressed genes, and black dots represent the constitutively expressed genes.

Analysis of highly variable expressed genes that contribute to cell-to-cell variation within bovine milk somatic cells revealed the most highly variable expressed genes: *CRCT1* (cystein rich C terminal 1), *S100A2* (S100 calcium-binding protein A2), caseins (*CSN1S1*, *CSN1S2*, *CSN2*, *and CSN3*) and whey proteins (*PAEP* and *LALBA*) (Figure 7).

## 4. Discussion

Traditionally, somatic cells in the milk are expected to belong to myo/epithelial mammary gland cells, different types of immune cells (lymphocytes, neutrophils, and macrophages) and stromal cells [35]. However, since precise markers for sub-differentiation of cell types in the mammary gland are not present in all mammalian species (agricultural species are not very well covered), the number of different cell types in the somatic cell fraction was normally underestimated. The analysis of bulk RNA transcripts from milk somatic cells revealed a very wide range of expressed genes and consequently indicated a wider range of cell types in the milk somatic cell fraction. Single-cell sequencing of human and mouse mammary somatic cells revealed a much wider range of cell types, which are present in the milk [20,25]. Our single-cell RNA sequencing analysis of bovine milk has revealed a cellular landscape of bovine milk somatic cells, highlighting a rich diversity of cell types pivotal for lactation, immune response, and tissue homeostasis. Similar to the findings of Becker et al. (2021) [15], our study also emphasizes the complexity of the mammary gland and reveals a broad spectrum of immune and epithelial cells. Becker et al. (2021) [15] identified 14 cell clusters in bovine milk, which were annotated as monocytes, CD4+ T cells, CD8+ T cells, B cells, macrophages, dendritic cells, NK cells, and epithelial cells. Despite using a consistent granularity parameter of 0.8 for clustering, our analysis revealed 21 distinct clusters. We identified an additional cluster, encompassing three subclusters of neutrophils. Neutrophils are a component of the defence mechanisms of the mammary gland and are naturally present in milk. However, their numbers increases in response to infection.

We applied an anchor-based workflow to integrate cells from two milk samples together. In the anchor-based approach, cross-dataset pairs of cells are identified, and cells that share a biological state are clustered together [31]. We identified cell subpopulations that are present in both datasets (Figure 2). By focusing on the mid-lactation in two Holstein Friesian cows, our study aims to capture a snapshot of the cellular mechanisms driving milk synthesis and secretion at a time when these processes are highly expressed. The mid-lactation phase is characterized by relatively stable milk production and provides a window to study the gene expression profiles of milk-producing cells without the effects of the ramp-up in early lactation or the wind-down phase towards the end of lactation.

Expression of genes, encoding caseins and whey proteins is characteristic of mammary epithelial cells. We identified cells producing caseins (*CSN1S1*, *CSN1S2*, *CSN2*, and *CSN3*) and whey proteins (*PAEP* and *LALBA*). Higher levels of caseins and whey proteins were detected in secretory alveolar cells. Detection of highly variable expressed genes allowed us to identify genes that strongly contribute to cell-to-cell variation within the cell population [36]. The most highly variable expressed genes in our samples were *CRCT1* (cystein-rich C terminal 1), *S100A2* (S100 calcium-binding protein A2), caseins (*CSN1S1*, *CSN1S2*, *CSN2*, *and CSN3*) and whey proteins (*PAEP* and *LALBA*). The gene *CRCT1* was associated with epidermal differentiation [37] and might allow insight into a possible differentiation pathway that plays a role in the differentiation process of the mammary gland. S100 calcium-binding protein A2 was identified in milk from cows with clinical mastitis [38], but later it was identified also in milk from healthy cows [39].

A recent analysis of single-cell transcriptomes in mice revealed important differences in gene expression between different cell types, which can significantly vary during the development of the mammary gland as well as in the course of lactation [21]. With the application of RNA sequencing for the study of milk transcriptome, the methodology for collecting biological samples became more and more important, especially for applications based on the single-cell sequencing approach. Since there is solid evidence that expression profiles and the proportion of different cell types in the mammary gland very much depend on the lactation stage, it is necessary to compare transcription profiles in different lactation stages, preferably in the same animal, because of the differences in the representation of cell types and differences in transcription profiles between animals [3,40]. Together with animal welfare arguments is the possibility of obtaining multiple samples from the same animal in the course of lactation a strong argument for a non-invasive sampling approach.

## 5. Conclusions

Our results shed light on the complex cellular landscape and gene expression profiles of bovine milk during mid-lactation. The identification of a considerably higher number of cell types in the milk somatic cell fraction compared to traditional expectations opens a new horizon for a more complex interpretation of the biological processes in the mammary gland.

## Figures and Tables

**Table 1:** Ten major marker genes were identified for each of 21 cell clusters and cluster annotations.

Cluster	Cluster Annotation	Marker Gene
0	CD8+ T cells	*GZMK*, *ENSBTAG00000027204*, *CD52*, *ENSBTAG00000010828*, *FAM162A*, *RGS1*, *CCL5*, *ENSBTAG00000034609*, *ENSBTAG00000000432*
1	CD4+ T cells	*UBD*, *ICOS*, *ENSBTAG00000055140*, *GUCY1B1*, *ENSBTAG00000027204*, *CD4*, *S100A13*, *NCR3*, *ENSBTAG00000034609*
2	Neutrophils	*GPR84*, *BASP1*, *PLAU*, *PLEK*, *MARCKS*, *IL1B*, *DMXL2*, *IL1RN*, *BATF3*
3	Progenitor cells	*CCL2*, *APOE*, *S100A2*, *CLDN4*, *CD9*, *CCL8*, *CST6*, *CRYAB*, *CRCT1*
4	Monocytes	*CD36*, *CTSS*, *CTSB*, *LIPA*, *CCL8*, *GRN*, *CNDP2*, *TREM2*, *CD9*
5	Monocytes	*EDN1*, *FCN1*, *LYZ*, *CD14*, *ARAF*, *TNF*, *CXCL3*, *BOLA-DRA*, *DEFB13*
6	Mast cells	*NUPR1*, *KIT*, *ENSBTAG00000055197*, *SPRY2*, *CTSW*, *ENSBTAG00000000144*, *CD7*, *TNFRSF9*, *ENSBTAG00000034609*
7	Macrophages	*FABP5*, *CD36*, *CTSB*, *APOE*, *CSTB*, *CNDP2*, *CTSZ*, *ATOX1*, *CD9*
8	Neutrophils	*BASP1*, *ENSBTAG00000048980 (Chemokine interleukin-8-like domain-containing protein)*, *IFITM3*, *CXCR1*, *GPR84*, *SELL*, *ENSBTAG00000034366*, *TGM3*, *S100A9*
9	B cells	*BLA-DQB*, *ENSBTAG00000013919*, *CD74*, *MS4A1*, *ENSBTAG00000055240*, *TNFRSF13C*, *CCR7*, *ENSBTAG00000009656*, *IRF4*
10	Progenitor cells	*TACSTD2*, *RASD1*, *ENSBTAG00000050919*, *DUSP5*, *EFNB2*, *ARC*, *KLF4*, *HSPA2*, *MAFB*
11	NK cells	*GNLY*, *ENSBTAG00000047449 (Saposin B-type domain-containing protein)*, *CD52*, *UBD*, *S100A13*, *PRF1*, *GPR183*, *ENSBTAG00000000144 (Ig-like domain-containing protein)*, *ENSBTAG00000055197 (Immunoglobulin C1-set domain-containing protein)*
12	Memory CD8+ T cells	*RUBCNL*, *UBD*, *CD52*, *BASP1*, *PLAU*, *ENSBTAG00000027204*, *PLEK*, *IL1RN*, *ENSBTAG00000034609*
13	Dendritic cells	*CCR7*, *GPR183*, *LY75*, *BLA-DQB*, *TAMALIN*, *PKIB*, *ENSBTAG00000013919*, *BOLA-DRA*, *FSCN1*
14	Monocytes	*PTGS2*, *CCL2*, *CD36*, *CCL8*, *CTSS*, *RUBCNL*, *EDN1*, *CXCL5*, *DEFB13*
15	Luminal progenitor cells	*CLU*, *CLDN3*, *CLDN4*, *CRYAB*, *KRT7*, *DSTN*, *WFDC2*, *KRT19*, *LTF*
16	Luminal cells	*CSN1S1*, *PAEP*, *CSN1S2*, *CSN3*, *GLYCAM1*, *LALBA*, *HSTN*, *SCGB1D*, *FABP3*
17	Luminal progenitor cells	*CRCT1*, *AGPAT2*, *CLDN3*, *KRT7*, *CLDN4*, *DSTN*, *S100A2*, *CRYAB*, *WFDC2*
18	Neutrophils	*TUBB4B*, *GADD45G*, *DDIT4*, *GADD45A*, *HSPH1*, *HSPA1A*, *IER5L*, *ZFAND2A*, *LRIF1*
19	CD4+ CD8+ T cells	*PCLAF*, *MKI67*, *STMN1*, *TOP2A*, *DUT*, *HMGB2*, *CENPF*, *TMPO*, *DNMT1*, *UBE2C*
20	Dendritic cells	*BOLA-DRA*, *ENSBTAG00000013919 (BOLA-DRB3)*, *ENSBTAG00000009656 (BOLA-DQA2)*, *CD74*, *C3H1orf54*, *BLA-DQB*, *CST3*, *ENSBTAG00000037605 (BOLA-DQA1)*, *BOLA-DMA*, *PLAC8A*

## Data Availability

The datasets presented in this study can be found in online repositories. Raw sequencing data were uploaded to European Nucleotide Archive (https://www.ebi.ac.uk/ena/browser/home). Accession number PRJEB73560.

## Supplementary Materials

The following supporting information can be downloaded at: https://www.mdpi.com/article/10.3390/genes15030349/s1, Figure S1: Assessment of cDNA amplification and library preparation integrity.
