# Supplementary material for: A Single-Cell Transcriptome of Bovine Milk Somatic Cells
Source: Genes (Basel). 2024 Mar 10;15(3):349. doi: 10.3390/genes15030349 (PMC10970057; doi:10.3390/genes15030349)
Supplement: Supplementary file 1 [file genes-15-00349-s001.zip › genes-2858795-supplementary.pdf]

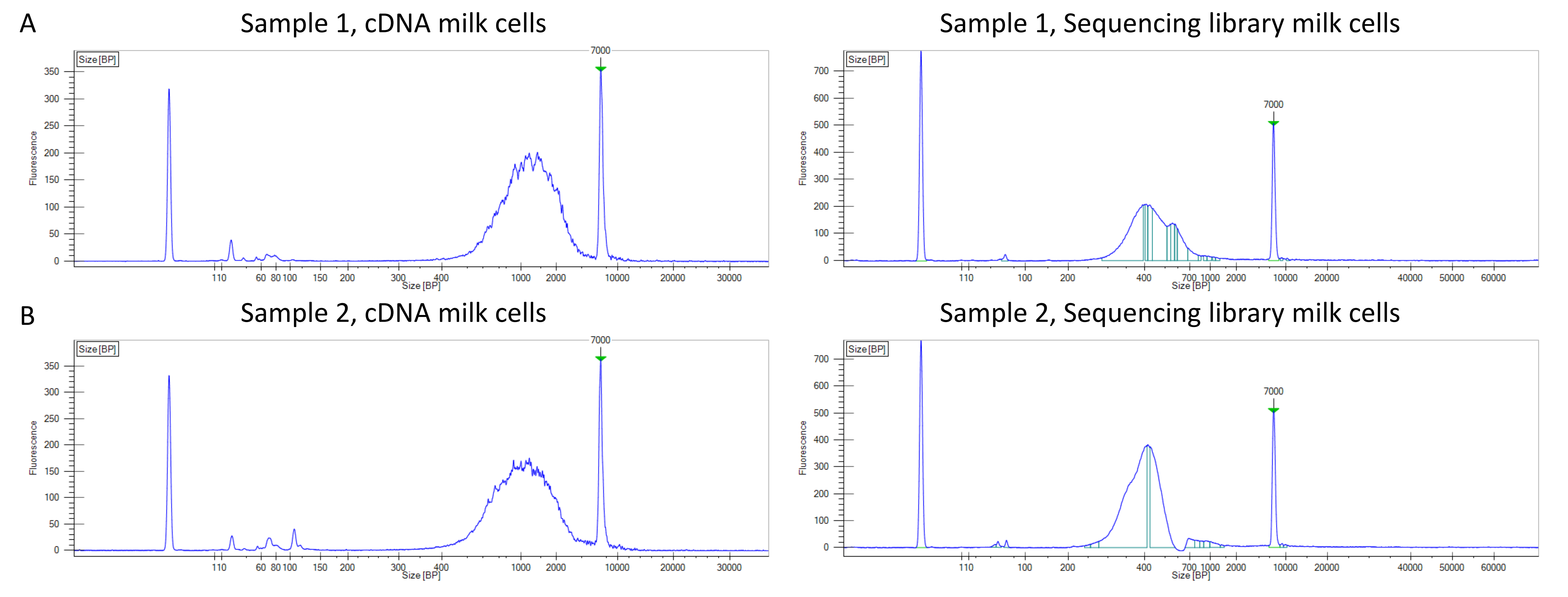

A
Sample 1, cDNA milk cells
Sample 1, Sequencing library milk cells
B
Sample 2, cDNA milk cells
Sample 2, Sequencing library milk cells
Size [BP]
Fluorescence
7000
0
50
100
150
200
250
300
350
400
500
600
700
110
60
80
1000
2000
10000
20000
30000
40000
50000
60000
